# Correlates of exercise motivation and behavior in a population-based sample of endometrial cancer survivors: an application of the Theory of Planned Behavior

**DOI:** 10.1186/1479-5868-4-21

**Published:** 2007-05-30

**Authors:** Kristina H Karvinen, Kerry S Courneya, Kristin L Campbell, Robert G Pearcey, George Dundas, Valerie Capstick, Katia S Tonkin

**Affiliations:** 1 Faculty of Physical Education and Recreation, University of Alberta, E-424 Van Vliet Center, Edmonton, Alberta, T6G 2H9, Canada; 2 Division of Radiation Oncology, Cross Cancer Institute, Edmonton, Alberta, Canada; 3 Department of Obstetrics and Gynecology, Faculty of Medicine, University of Alberta, Edmonton, Alberta, Canada; 4 Division of Medical Oncology, Cross Cancer Institute, Edmonton, Alberta, Canada

## Abstract

**Background:**

Despite evidence of the benefits of exercise in cancer survivors, exercise participation rates tend to decline after treatments. Few studies have examined the determinants of exercise in less common cancer sites. In this study, we examined medical, demographic, and social cognitive correlates of exercise in endometrial cancer survivors using the Theory of Planned Behavior (TPB).

**Methods:**

A mailed survey was completed by 354 endometrial cancer survivors (1 to 10 years postdiagnosis) residing in Alberta, Canada. The study was cross-sectional. Exercise behavior was assessed using the Godin Leisure Time Exercise Questionnaire and the TPB constructs were assessed with standard self-report scales. Multiple regression analyses were used to determine the independent associations of the TPB constructs with intention and behavior.

**Results:**

Chi-square analyses indicated that marital status (*p *= .003), income level (*p *= .013), and body mass index (BMI) (*p *= .020) were associated with exercise. The TPB explained 34.1% of the variance in exercise behavior with intention (*β *= .38, *p *< .001) and self-efficacy (*β *= .18, *p *= .029) being independent correlates. For intention, 38.3% of the variance was explained by the TPB with self-efficacy (*β *= .34, *p *< .001) and affective attitude (*β *= .30, *p *< .001) being the independent correlates. The TPB mediated the associations of marital status and BMI with exercise but not income level. Age and BMI moderated the associations of the TPB with intention and behavior.

**Conclusion:**

The TPB may be a useful framework for understanding exercise in endometrial cancer survivors. Exercise behavior change interventions based on the TPB should be tested in this growing population.

## Background

Exercise has received attention as a means of ameliorating physical, functional, and emotional quality of life issues in cancer survivors [1]. Despite the many documented benefits of exercise in cancer survivors, exercise participation rates are relatively low [2,3] suggesting the importance of identifying the determinants of exercise in this group. Several studies have explored exercise motivation in cancer survivors [4]. Most studies have focused on the most common cancers such as breast [5], prostate [5], and colorectal [6], however, recent studies have also examined less common cancers such as non Hodgkins lymphoma [3], multiple myeloma [7] and brain cancer [8]. The results have indicated that the determinants of exercise may vary depending on the cancer survivor group. Consequently, different cancer survivor groups may present unique determinants of exercise, and generalizations among cancer survivor groups may not be warranted. Endometrial cancer survivors are unique because they have a tendency to be obese [9], often suffer from other co-morbidities [10], and have a number of late and chronic side-effects from treatments such as urinary incontinence, diarrhea, and mood disturbances [11] that might affect exercise motivation and behavior.

Many studies to date have applied the Theory of Planned Behavior (TPB) for understanding exercise motivation in cancer survivors and other populations [12] . The TPB may be a particularly relevant framework to use in cancer survivors because it includes personal (attitude), normative (subjective norm), and control (perceived behavioral control) factors that all may be influenced by treatment-related side-effects and co-morbidities. The TPB postulates that intention (motivation and plans) is the immediate determinant of behavior. Intention, in turn, is determined by subjective norm (perceived social pressure to perform the behavior), attitude (positive or negative evaluation of the behavior), and perceived behavioral control (PBC; confidence and control over performing the behavior). Moreover, subjective norm is based upon normative beliefs (specific individuals that may approve or disapprove of the behavior), attitude is based upon behavioral beliefs (specific perceived advantages and disadvantages of participating in the behavior), and PBC is based upon control beliefs (specific barriers and opportunities the individual has for performing the behavior).

Each TPB construct (i.e., attitude, subjective norm, PBC) is considered to be a higher order structure that is composed of two lower order components [13]. Attitude consists of affective (enjoyment of exercise) and instrumental (perceived benefits of exercise) components, subjective norm is comprised of descriptive (perception that important others exercise) and injunctive (perception that important others approve of exercise) components, and PBC consists of self-efficacy (confidence in ability to exercise) and perceived control (perceived control over exercise) components. Some theorists have proposed that the multidimensional testing of the lower order structures (i.e., affective and instrumental attitudes, descriptive and injunctive norms, self-efficacy and perceived control) instead of the aggregated higher order structures results in better model fit and higher explained variance [14-16].

In addition to testing the traditional TPB constructs, we were also interested in examining how medical and demographic variables correlate with exercise and/or moderate the associations between the TPB and exercise in endometrial cancer survivors. Research in other populations has indicated that some medical and demographic variables, like age [17], body mass index [18], and socioeconomic status [19], have been found to be associated with exercise participation and may influence exercise determinants.

To date, no study has examined individual motivational determinants of exercise in endometrial cancer survivors, even though endometrial cancer ranks as the fourth most common cancer in women and has a five-year relative survival rate of 84% [20,21]. Furthermore, endometrial cancer survivors have a higher rate of obesity and co-morbidities [9], and consequently may be a population who could benefit considerably from exercise.

Our primary purpose was to examine correlates of exercise intention and behavior in endometrial cancer survivors. Based on the theoretical tenets of the TPB, we hypothesized that intention would be the most important correlate of exercise behaviour and that attitude, subjective norm, and perceived behavioral control would each be independent correlates of exercise intention. We also expected that the TPB would mediate any associations between any demographic and medical variables associated with exercise. Our secondary purpose was to explore potential medical and demographic moderators of these associations.

## Methods

### Sample and procedures

The Alberta Cancer Board and the University of Alberta provided ethical approval for the study. All contact information was obtained through the Alberta Cancer Registry and involved a list of all endometrial cancer survivors diagnosed in Alberta, Canada between November, 1994 and June, 2003. Participants were eligible for the study if they were: (a) over the age of 18 years, (b) English speaking, (c) approved for contact by a family physician or primary oncologist (as required by our ethics boards), and (d) had a histologically confirmed diagnosis of endometrial cancer. We first contacted each survivor's physician or oncologist to get approval to contact their patient. Once physician/oncologist approval was obtained, endometrial cancer survivors were mailed a questionnaire package and asked to return the questionnaire plus one copy of the consent form. A slightly modified version of the Total Design Method [22] was used that consisted of: (a) mailing of the initial questionnaire package, (b) mailing of a postcard reminder two weeks later, and (c) mailing of a second questionnaire package four weeks later. Other methods known to improve survey response rates were also used, such as postage paid business reply envelopes, personalized cover letters with original signatures, colored paper questionnaires, assurances of confidentiality, and institution sponsorship [23]. All participants gave consent for the researchers to utilize their medical data. The study was conducted between March and May of 2004.

### Measures

Marital status, annual income, employment status, education level, height, and weight were obtained by self-report. Age, time since diagnosis, tumor grade, disease stage, and treatment type were collected by both self-report and the registry records. Registry data were used and, if unavailable, self-report data were used as a supplement.

A modified version of the Leisure Score Index (LSI) from the Godin Leisure Time Exercise Questionnaire (GLTEQ) [24] was used to assess exercise behavior. The GLTEQ is considered one of the most reliable measures of self-reported exercise and it is easy to administer and brief. The GLTEQ assesses average frequency and duration of exercise at three levels of intensity: mild (minimal effort, no perspiration), moderate (not exhausting, light perspiration), and strenuous (heart beats rapidly, sweating). We did not use the mild minutes for our calculations, but included the category in the questionnaire to ensure that participants did not report mild exercise minutes in the moderate intensity category [25]. Our interest in only moderate and vigorous exercise minutes is based on public health recommendations that suggest that moderate-to-vigorous intensity activity is required to obtain health benefits [26]. We calculated weekly exercise minutes separately for moderate and strenuous exercise as well as a combined score for moderate plus strenuous exercise. We then categorized participants as: (a) meeting public health exercise guidelines (≥ 60 minutes of strenuous or ≥ 150 minutes of moderate plus strenuous exercise per week), (b) not meeting public health exercise guidelines but accumulating some moderate-to-strenuous exercise minutes or (c) not reporting any moderate-to-strenuous exercise [26]. This trichotomous ordinal variable was used as our exercise behavior variable because of the highly skewed distribution of exercise behavior using the continuous scale. An independent evaluation of the GLTEQ reported its reliability and validity to compare favorably to nine other self-report measures of exercise based on test-retest scores, an objective activity monitor (i.e., accelerometer), and fitness indices. The LSI showed a one-month test-retest reliability of .62 and concurrent validity coefficients of .32 with the accelerometer, .56 with VO_2max_, and -.43 with % body fat [27].

The Theory of Planned Behavior (TPB) constructs were assessed based on Ajzen's recommendations [12,13]. At the beginning of the TPB scales, we provided the following definition of regular exercise as: "vigorous physical activity (e.g., sweating, heart beats fast) performed for at least 3 times per week for 20 minutes or more, or moderate physical activity performed at least 5 times per week for 30 minutes or more".

Intention was measured by two items on 7-point Likert scales ranging from 1 (strongly disagree) to 7 (strongly agree). The first item was "I intend to exercise for at least 30 minutes, 5 days per week at a moderate intensity" and the second item was "I intend to exercise for at least 20 minutes, 3 days per week at a vigorous intensity". Internal consistency (α) for these two items was .70. The intention variable was the mean of the two items.

Attitude was assessed by seven items measured on 7-point bipolar adjective scales. These items assessed both instrumental (harmful-beneficial, foolish-wise, useless-useful, good-bad) and affective (unenjoyable-enjoyable, boring-fun, unpleasant-pleasant) components of attitude [13]. Verbal descriptors were extremely (points 1 and 7), quite (points 2 and 6), and slightly (points 3 and 5). The stem that preceded the items was "For me, exercising regularly over the next month would be". Internal consistencies (α) for the instrumental and affective subscales were .92 and .89, respectively. We scored instrumental and affective attitude separately.

Subjective norm was determined using three items on a 7-point Likert scale ranging from 1 (strongly disagree) to 7 (strongly agree). These items assessed the injunctive component of subjective norm. The three items were "Most people who are important to me (1) think I should, (2) would encourage me to, and (3) would support me, exercising regularly over the next month". Internal consistency (α) for this scale was .91.

Perceived behavioral control was assessed by four items on a 7-point Likert scale that tapped self-efficacy and controllability [13]. The three items that comprised self-efficacy were: "If you were really motivated, (1) exercising over the next month would be.... " (1 = extremely hard to 7 = extremely easy), (2) "how confident are you that you could exercise regularly over the next month" (1 = not at all confident to 7 = extremely confident), and (3) "I could easily exercise regularly over the next month" (1 = strongly disagree to 7 = strongly agree). The item that comprised the controllability component was "If you were really motivated, how much control do you feel you would have over exercising regularly over the next month" (1 = very little control to 7 = complete control). Internal consistency (α) of the self-efficacy scale was .86. Self-efficacy and perceived control were scored separately.

Underlying beliefs were assessed using open ended questions. Participants were asked to list: (1) " ...the main advantages of participating in regular exercise" (behavioral beliefs), (2) factors that " ...make it difficult for you to exercise regularly" (control beliefs), and (3) " ...people or groups who would approve or disapprove of you exercising" (normative beliefs).

### Analysis

Data were analyzed using SPSS 13 (SPSS Inc., Chicago, Ill). Descriptive statistics were calculated to determine the distribution of the variables. Chi-square tests were used to determine the association between exercise and demographic (i.e., age, marital status, education level, income level, employment status) and medical variables (i.e., adjuvant treatment, body mass index, months since diagnosis). Pearson correlation coefficients were calculated to determine the bivariate associations between the TPB constructs. Forced entry multiple regression procedures were used to test the primary hypotheses. Two separate multiple regression analyses were conducted to determine which of the TPB constructs (i.e., affective attitude, instrumental attitude, subjective norm, perceived control, self-efficacy) and any univariate significant demographic and medical variables independently correlated with each of the criterion variables (i.e., exercise behavior, exercise intention). Medical and demographic variables were dichotomized for these analyses (Table 1). For medical and demographic variables that were found to be associated with exercise but mediated by the TPB, follow-up independent t-tests were conducted that compared scores on the TPB constructs based on different levels of the relevant medical or demographic variable.

**Table 1:** Percentage of participants in each exercise category based on demographic and medical variables.

Variable	Completely Sedentary	Insufficiently Active	Meeting Guidelines	χ ^2^	p
Age					
< 65 years (n = 181)	39.8%	24.9%	35.4%	2.8	.242
≥ 65 years (n = 173)	48.6%	20.2%	31.2%		
Marital Status					
Married/Common Law (n = 245)	38.4%	26.1%	35.5%	11.4	.003
Not Married (n = 109)	56.9%	14.7%	28.4%		
Education					
Completed High School or Less (n = 168)	48.8%	23.2%	28.0%	4.4	.109
Some Post Secondary or More (n = 186)	39.8%	22.0%	38.2%		
Income					
< $40,000/year (n = 169)	50.9%	23.1%	26.0%	8.6	.013
≥ $40,000/year (n = 185)	37.8%	22.2%	40.0%		
Employment					
Working (n = 112)	36.6%	27.7%	35.7%	4.2	.121
Not working (n = 242)	47.5%	20.2%	32.2%		
Body Mass Index					
Non-obese (n = 215)	38.1%	24.7%	37.2%	7.9	.020
Obese (n = 139)	53.2%	19.4%	27.3%		
Months Since Diagnosis					
< 60 months (n = 221)	45.2%	21.7%	33.0%	0.4	.817
≥ 60 months (n = 133)	42.1%	24.1%	33.8%		
Adjuvant Therapy					
Yes (n = 188)	46.8%	20.2%	33.0%	1.7	.426
No (n = 166)	41.0%	25.3%	33.7%		

Subsequent regression analyses were conducted in order to test for the potential moderating effects of several medical and demographic variables (i.e., age, BMI, months since diagnosis, adjuvant treatment) on the associations among the TPB. We created ordinal or dichotomous variables for these analyses. Age was divided into < 60 years (*n *= 119), 60 – 69 years (*n *= 133), and ≥ 70 years (n = 102) and BMI was divided into healthy weight (< 25; *n *= 102), overweight (25 – 29.9; *n *= 113), and obese (≥ 30; *n *= 139). Months since diagnosis was divided into < 60 months (*n *= 221), and ≥ 60 months (*n *= 133) based on the approximate median of the number of months since diagnosis as well as a commonly accepted cut-point for a "long term survivor" (i.e., five-year survivor) [21]. Treatment was categorized as having received any adjuvant treatment (i.e., chemotherapy, radiation therapy or hormone therapy; *n *= 183) versus no adjuvant treatment (*n *= 164). Interaction terms were created by multiplying the potential moderator (e.g., age) by each of the TPB constructs (e.g., intention by age, instrumental attitude by age, etc.).

For the moderator analyses, hierarchical forced entry multiple regression procedures were conducted using the procedures outlined by Baron and Kenny [28]. In the first analysis, we regressed exercise behavior on exercise intention, self-efficacy, and perceived control, followed by the particular moderator (e.g., age), and then the three interaction terms (e.g., age by intention). In the second analysis, exercise intention was regressed on the five TPB constructs followed by the particular moderator, and finally the five interaction terms [29]. Although the study design was cross-sectional, the moderators we tested (i.e., demographic and medical variables) are antecedents to the TPB constructs and thus qualify as moderators for our analyses as recommended by Kraemer et al. [30]. We also repeated the analyses with each of the independent variables mean centered (i.e., the individual score subtracted from the group mean) to reduce any potential effects of multicollinearity [29] but did not find any meaningful differences. We present the results of the uncentered analyses.

## Results

Flow of participants through the study has been presented elsewhere [31]. In summary, of the 769 eligible endometrial cancer survivors to whom we mailed questionnaire packages, 386 returned the completed questionnaire (response rate = 50.2%). For the present analyses, we had 354 individuals provide evaluable data since 32 of the original participants were missing data on one or more of the TPB variables. We compared responders, non-responders, and non-approvals (by their physicians) in terms of medical and demographic variables and found that the only significant difference (*p *< .001) was that fewer non-approvals had received radiation therapy (24.6%) compared to responders (49.3%) and non-responders (48.7%).

Details of demographic and medical variables are reported elsewhere [31]. In brief, 44% (*n *= 156) of participants were not exercising at all, 23% (*n *= 80) were not meeting public health exercise guidelines but were accumulating some moderate-to-strenuous exercise minutes, and 33% (*n *= 118) were meeting public health exercise guidelines. The mean age of the sample was 64.5 years (*SD *= 10.6), 69% were married, 38% had completed university/college, and 63% were retired. The mean number of months since diagnosis was 52 (*SD *= 32; range: 9–115), 54% had disease stage I, 18% had stage II, 9% had stage III, and 18% were ambiguous (likely stage I). For treatment, 99% had undergone surgery, 48% had received radiation therapy, 8% had received chemotherapy, and 7% had received hormone therapy. Mean BMI was 29.3 (*SD *= 6.6).

Table 1 displays differences in exercise levels based on the demographic and medical variables. Significant differences were found in exercise level based on marital status (χ^2 ^= 11.4, *p *= .003), income (χ^2 ^= 8.6, *p *= .013) and BMI (χ^2 ^= 7.9, *p *= .020). Descriptive statistics of the TPB variables indicated that participants, on average, had neutral intentions to exercise regularly over the next month. Participants indicated quite positive instrumental attitudes and slightly positive affective attitudes. On average, participants moderately agreed that important others in their life would encourage and support their exercise efforts. They also believed exercising regularly would be slightly easy, and felt they had moderate control over exercise (Table 2).

**Table 2:** Descriptive Statistics and Correlations for the Theory of Planned Behavior (N = 354).

Variable	2.	3.	4.	5.	6.	7.	*M*	*SD*
1. Exercise Behavior	.53*	.42*	.41*	.38*	.49*	.44*	0.89	0.87
2. Intention		.46*	.53*	.34*	.56*	.47*	4.14	1.86
3. Instrumental Attitude			.53*	.65*	.66*	.64*	6.17	1.02
4. Affective Attitude				.33*	.54*	.47*	5.25	1.29
5. Subjective Norm					.53*	.52*	5.95	1.17
6. Self-efficacy						.84*	5.40	1.42
7. Perceived Control							5.27	1.67

*p < .001

*M *= mean

*SD *= standard deviation

### Correlations among Theory of Planned Behavior constructs

Bivariate correlations among the TPB constructs and exercise behavior were positive and significant (Table 2). Intention had the strongest correlation with exercise behavior (*r *= .53, *p *< .001). Self-efficacy (*r *= .56, *p *< .001) and affective attitude (*r *= .53, *p *< .001) had the strongest correlations with intention.

### Multivariate tests of the Theory of Planned Behavior

Results of the multiple regression procedures (Table 3) indicated that the TPB explained 34.1% of the variance in exercise behavior with intention (*β *= .38, *p *< .001) and self-efficacy (*β *= .18, *p *= .029) providing independent associations. When the significant demographic and medical variables (i.e., marital status, income level and BMI) were added, an additional 2.3% of the variance in exercise behavior was explained by the model, with income level independently correlating with exercise behavior (*β *= .11, *p *= .013). Marital status and BMI were not found to independently correlate with exercise behavior, but rather were found to be mediated by the TPB, evident by them not being significant after controlling for the TPB constructs in the model. Subsequent independent t-tests that compared the demographic/medical subgroups on scores on the TPB variables indicated which TPB construct mediated that particular demographic/medical variable. Those who were married reported significantly higher scores on subjective norm (*t *= 2.8, *p *= .006) and instrumental attitude (*t *= 2.0, *p *= .042) compared to those survivors who were not married. Those who were obese reported significantly lower affective attitudes (*t *= 4.5, *p *< .001), self-efficacy (*t *= 2.7, *p *= .006) and perceived control (*t *= 2.1, *p *= .036) and higher subjective norms (*t *= 2.1, *p *= .037) compared to those who were healthy weight/overweight.

**Table 3:** Hierarchical Forced Entry Multiple Regression Analyses.

*Exercise Behavior on Intention, Perceived Control, and Self-Efficacy*					
	*F*_change_	*df*	*R*^*2*^_change_	*β*^*1*^	*β*^*2*^

(Block #1)	60.5	3,350	.34		
Intention				.38***	.38***
Self-efficacy				.18*	.14
Perceived Control				.11	.13
(Block #2)	4.7	6,347	.03		
Marital status					.04
Income					.13**
BMI					-.07
Total Model	60.5	3,350	.34		

*Exercise Intention on the Theory of Planned Behavior*					

	*F*	*df*	*R*^*2*^	*β*	

Instrumental Attitude				.06	
Affective Attitude				.30***	
Subjective Norm				.02	
Self-Efficacy				.36***	
Perceived Control				-.02	
Total Model	43.12	5,348	.38		

* = *p *< .05

** = *p *< .01

*** = *p *< .001

*F *= F ratio

*df *= degrees of freedom

*R*^2 ^= coefficient of determination

β^1–2 ^= *standardized regression coefficients for equations #1 and #2*

For intention, 38.3% of the variance was explained, with self-efficacy (*β *= .34, *p *< .001) and affective attitude (*β *= .30, *p *< .001) providing independent associations.

### Medical and demographic moderators of the TPB

For exercise behavior, we found no significant moderating effects of BMI, months since diagnosis, or treatment. We did, however, find a significant interaction of age with intention (*p *= .001) and perceived control (*p *= .001; Table 4). Subsequent separate regression analyses of the three age categories revealed that exercise intention was independently associated with exercise behavior in survivors under the age of 60 years (*β *= .55, *p *< .001) and between the ages of 60 and 70 (*β *= .45, *p *< .001) but not in the over 70 age group (*β *= .09, *p =*.402) whereas perceived control was the only independent correlate of exercise behavior in the oldest age category (*β *= .39, *p *= .003).

**Table 4:** Hierarchical Forced Entry Multiple Regression of Exercise Behavior on Intention, Perceived Control, Self-Efficacy, Age, and Interactions with Age.

	*F*_change_	*df*	*R*^*2*^_change_	*β*^*1*^	*β*^*2*^	*β*^*3*^
(Block #1)	60.5	3,350	.34			
Intention				.38***	.39***	.79***
Self-efficacy				.18*	.17*	.31
Perceived Control				.11	.11	-.55*
(Block #2)	3.7	1,349	.01			
Age					-.08	-.33
(Block #3)	7.4	3,346	.04			
Age*Intention						-.57**
Age*Self-efficacy						-.17
Age*Perceived Control						.95**
Total Model	31.3	7,346	.39			

* = *p *< .05

** = *p *< .01

*** = *p *< .001

*F *= F ratio

*df *= degrees of freedom

*R*^2 ^= coefficient of determination

*β*^1–3 ^= standardized regression coefficients for equations #1 to #3

For exercise intention, we found no moderating effects of age, months since diagnosis, or treatment but we did find a significant interaction for BMI with instrumental attitude (*p *= .002) and self-efficacy (*p *= .001; Table 5). Follow up regression analyses for each of the three separate BMI categories indicated that instrumental attitude was independently associated with intention in healthy weight survivors (*β *= .61, *p *< .001), but not in overweight (*β *= .04, *p *= .718) or obese survivors (*β *= -.05, *p *= .635). Conversely, self-efficacy was independently associated with exercise intention in obese survivors (*β *= .57, *p *< .001), but not in healthy weight (*β *= -.12, *p *= .490) or overweight survivors (*β *= .25, *p *= .103).

**Table 5:** Hierarchical Forced Entry Multiple Regression of Exercise Intention on the Theory of Planned Behavior, BMI, and Interactions with BMI

	*F*_change_	*df*	*R*^*2*^_change_	*β*^*1*^	*β*^*2*^	*β*^*3*^
(Block #1)	43.1	5,348	.38			
Instrumental Attitude				.06	.06	.76**
Affective Attitude				.30***	.31***	.13
Subjective Norm				.02	.01	-.05
Self-efficacy				.36***	.36***	-.41
Perceived Control				-.02	-.02	.17
(Block #2)	0.8	1,347	.00		.04	.31
BMI						
(Block #3)	4.1	5,342	.04			
BMI*Instrumental Attitude						-1.60**
BMI*Affective Attitude						.24
BMI*Subjective Norm						.13
BMI*Self-efficacy						1.20**
BMI*Perceived Control						-.23
Total Model	22.4	11,342	.42			

* = *p *< .05

** = *p *< .01

*** = *p *< .001

*F *= F ratio

*df *= degrees of freedom

*R*^2 ^= coefficient of determination

*β*^1–3 ^= standardized regression coefficients for equations #1 to #3

### Most common accessible beliefs

The five most commonly reported beliefs for each belief category were as follows. Behavioral beliefs: (1) lose weight, (2) feel better about one's self, (3) keep in shape, (4) improve strength/tone muscles, and (5) improve cardiovascular health. Control beliefs: (1) poor health, (2) lack of time, (3) poor weather conditions, (4) injury, and (5) fatigue/lack of energy. Normative beliefs: (1) doctor, (2) spouse, (3) family, (4) children, and (5) friends (Table 6).

**Table 6:** Most Common Behavioral, Control, and Normative Beliefs of Endometrial Cancer Survivors Concerning Regular Exercise (N = 354).

	n	% survivors^1^	% respondents^2^
Most Common Behavioral Beliefs (Advantages)			
Lose weight	127	35.9	45.8
Feel better about self	99	28.0	35.7
Keep in shape	92	26.0	33.2
Improves strength/tones muscles	62	17.5	22.4
Improve cardiovascular health	51	14.4	18.4
Improves mental health	41	11.6	14.8
Improves flexibility	17	4.8	6.1
Improves breathing	16	4.5	5.8
Aids in recovery from injuries	10	2.8	3.6
			
Most Common Control Beliefs (Barriers)			
Poor health	56	15.8	25.9
Lack of time	52	14.7	24.1
Poor weather conditions	47	13.3	21.8
Injury	39	11.0	18.1
Fatigue/lack of energy	24	6.8	11.1
Lack of access to facilities	23	6.5	10.6
Lack of motivation	17	4.8	7.9
Frequent disruptions to schedule	12	3.4	5.6
Family demands	11	3.1	5.1
			
Most Common Normative Beliefs (Approve)			
Doctor	152	42.9	52.1
Spouse	132	37.3	45.2
Family	111	31.4	38.0
Children	110	31.1	37.7
Friends	79	22.3	27.1
Everyone	44	12.4	15.1

^1 ^Percentage of responses from all participants (N = 354).

^2 ^Percentage of responses from participants who answered the question.

## Discussion

In support of our primary hypothesis, the TPB accounted for 31% of the variance in exercise behavior, and 38% of the variance in exercise intentions. These findings are comparable to a meta-analysis that found, in exercise studies of the general population, the TPB accounted for 27.4% of the variance in behavior and 44.5% of the variance in intention [32]. In the literature on cancer survivors, the TPB has generally explained 14% to 35% of the variance in exercise behavior, and 23% to 68% of the variance in exercise intention [4].

An examination of the correlates of exercise behavior and motivation from the present study suggests endometrial cancer survivors show some similarities to other cancer survivor groups but also several important differences. Consistent with all previous research with cancer survivors, our study found that intention was an independent correlate of exercise behavior [4]. Self-efficacy was also found to an independent correlate of exercise behavior, a finding that was similar to several other studies that found PBC to be an independent correlate of exercise behavior [2,6].

We also found that affective attitude and self-efficacy were the independent correlates of intention in our overall analysis. These results are similar to other studies of non-clinical populations and cancer survivors that suggest attitude and PBC are the key correlates of intention [4,32]. Other research has also specifically indicated affective attitude to be a significant correlate of intention in cancer survivors [3]. The finding that subjective norm was not an independent correlate of intention in the present study, however, is different from other research [3]. This may be because the majority of participants in the present study were overweight or obese and therefore may be accustomed to hearing advice from others to exercise more. For example, physicians are more likely to offer advice on exercising and weight loss to obese patients than to those who are non-obese [33]. These women may, as a result, be less responsive to advice from others to exercise since they have heard it so much in the past. We did not, however, assess descriptive norm, which may have correlated with intention.

Also in support of our hypothesis, we found that the TPB mediated the associations that marital status and BMI had with exercise. Specifically, survivors who were not married and who were obese had the lowest exercise participation. Interestingly, married survivors indicated significantly higher subjective norms than survivors who were not married, a finding that is in line with past research that suggests the importance of spousal support in exercise adherence [34]. Consistent with past research on exercise in the obese in the general population [18,35,36], we found significantly lower scores on affective attitudes, self-efficacy and perceived control in obese survivors, indicating that they find exercise to not be as enjoyable and have feel less confidence and control over exercising regularly compared to health weight/overweight survivors. Interestingly, and contrary to our hypotheses, we found that income independently correlated with exercise after controlling for the TPB. Although a relationship between income and exercise has been documented consistently in the general population [19], we had expected that the TPB constructs would account for this association in endometrial cancer survivors.

From our exploratory analyses, we found that age and BMI moderated the associations of the TPB. Interestingly, we found that control constructs (self-efficacy and perceived control) were the most important correlates of exercise and exercise intention in older survivors and obese survivors, but not younger or healthy/overweight survivors. These findings are in line with Bandura's Social Cognitive Theory that states that control issues are most relevant under challenging conditions [37]. The number of co-morbidities in older compared to younger survivors may make it more challenging to exercise (e.g., health problems, injuries, pain) while obstacles such as excess body weight and other health conditions may hinder self-confidence for exercise in obese survivors.

This study also documented the underlying beliefs that endometrial cancer survivors have about exercise. These findings are important since they: (a) can be used to design TPB questionnaires that include belief-based measures for endometrial cancer survivors and (b) provide an understanding of attitudes, subjective norms, and perceived behavioral control in this population that can be targeted in interventions. The finding that weight loss is the most commonly reported behavioral belief is consistent with the high rate of obesity in this population, as well as previous research which suggests weight loss is a key motivator for exercise in women [38]. Additionally, the key behavioral beliefs appear to be primarily about physical health benefits. These findings are in line with previous research that has found health to be the most common motivator for physical activity [39].

The control beliefs reported in the present study are similar to those of other research with cancer survivors, which also cite lack of time, fatigue, and various health related problems as important barriers [40]. Poor health was, however, a novel control belief reported in the present study. This barrier may be paramount for this population because of the high obesity rate, and the greater rate of co-morbidities attached to obesity, such as diabetes, hypertension, cardiovascular disease, and other cancers [41]. Moreover, poor health has been found to be a major barrier to exercise adoption in older adults in general [42].

The present study has a number of strengths and limitations. The main strengths of this study include being the first study to examine exercise motivation and behavior in endometrial cancer survivors, testing a validated theoretical framework, using validated TPB and exercise measures, and obtaining a large and representative sample of endometrial cancer survivors in the Alberta population. The main weaknesses of our study are the cross-sectional study design and the self-report measure of exercise. The cross-sectional design means that the data on exercise behavior are actually retrospective rather than prospective. A recent study, however, has found that exercise behavior in the past month correlates strongly with exercise behavior measured prospectively, suggesting that cross-sectional studies may only slightly overestimate the association between social cognitive variables and exercise behavior [43]. Another limitation is that we did not measure descriptive norm, only injunctive norm, so our subjective norm variable is composed only of this subscale.

## Conclusion

The findings from this study have a number of practical implications that may be useful for guiding future intervention studies. Exercise intervention studies in endometrial cancer survivors should be tested to see if strengthening exercise intentions in younger survivors and improving perceived control can reduce the intention-behavior gap. Exercise intentions in endometrial survivors may also be optimally promoted by developing programs that are enjoyable and fun and include strategies to improve self-efficacy for exercise. Moreover, intervention studies aimed at healthy weight survivors might be most effective if they focused on the potential benefits of exercise while those targeting obese survivors may be most effective if they improve exercise self-efficacy. Researchers and practitioners should also be aware that differences in exercise level may occur based on medical and demographic variables, such as marital status, income and BMI, in endometrial cancer survivors, but that many of these associations may be mediated by the constructs of the TPB. Future research will be able to determine the relative importance of the underlying beliefs that we identified in the present study and these should be the primary targets for interventions.
